# Brazilian Propolis Antileishmanial and Immunomodulatory Effects

**DOI:** 10.1155/2013/673058

**Published:** 2013-05-16

**Authors:** Suelen Santos da Silva, Graciele da Silva Thomé, Allan Henrique Depieri Cataneo, Milena Menegazzo Miranda, Ionice Felipe, Célia Guadalupe Tardeli de Jesus Andrade, Maria Angélica Ehara Watanabe, Gilce Maria Piana, José Maurício Sforcin, Wander Rogério Pavanelli, Ivete Conchon-Costa

**Affiliations:** ^1^Department of Science Pathology, Londrina State University (UEL), 86057-970 Londrina, PR, Brazil; ^2^Department of General Biology, Laboratory of Electron Microscopy and Microanalysis, Londrina State University (UEL), 86057-970 Londrina, PR, Brazil; ^3^Department of Microbiology and Immunology, Biosciences Institute (UNESP), 18618-970 Botucatu, SP, Brazil

## Abstract

The antileishmanial and immunomodulatory effects of propolis collected in Botucatu, São Paulo State, Brazil, were evaluated in *Leishmania (Viannia) braziliensis* experimental infection. The antileishmanial effect of propolis on promastigote forms was verified by reducing growth and by promoting morphologic alterations observed by scanning electron microscopy. In *in vitro* immunomodulatory assays, macrophages were pretreated with propolis and then infected with *L. (V.) braziliensis*. *In vivo*, supernatants from liver cells and peritoneal exudate of BALB/c mice pretreated with propolis and infected with *Leishmania* (10^7^/mL promastigotes) were collected, and TNF-*α* and IL-12 were measured by ELISA. Macrophages incubated with propolis showed a significant increase in interiorization and further killing of parasites. An increased TNF-*α* production was seen in mice pretreated with propolis, whereas IL-12 was downregulated during the infection. In conclusion, Brazilian propolis showed a direct action on the parasite and displayed immunomodulatory effects on murine macrophages, even though the parasite has been reported to affect the activation pathways of the cell. The observed effects could be associated with the presence of phenolic compounds (flavonoids, aromatic acids, and benzopyranes), di- and triterpenes, and essential oils found in our propolis sample.

## 1. Introduction

Leishmaniasis is caused by several species of the protozoa *Leishmania*. The severity of the disease may vary from cutaneous, mucosal, and diffuse cutaneous to visceral infections. The protozoa species and the host immune response determine the clinical forms of this disease [1]. In murine experimental models of cutaneous leishmaniasis with *Leishmania major *(*L. major*), the resistance to leishmaniasis is associated with a predominant Th1 response, while the susceptibility of some mouse strains (such as BALB/c) is assigned to a Th2 response [2]. Nevertheless, the evaluation of injuries caused by *Leishmania (Leishmania) amazonensis* (*L. amazonensis*) and *Leishmania (Viannia) braziliensis *(*L. braziliensis*) in inbred mice showed a different profile of susceptibility and resistance than that proposed to *L. major *infection model. It has been demonstrated that most strains of mice are genetically susceptible to *L. amazonensis* whereas for *L. braziliensis* the most strains of mice tested, including the BALB/c, have only self-healing lesions in the skin [3, 4].

Leishmaniasis chemotherapy is based on the use of pentavalent antimonial drugs. Other drugs, such as pentamidine and amphotericin B, have been adopted as alternative drugs; however, these drugs present severe side effects, including parasite resistance and long-term treatment [5, 6]. The discovery of new compounds with antileishmanial and immunomodulatory properties is essential for the development of new alternative to leishmaniasis therapy. Sforcin and Bankova pointed out that propolis have a great potential for the development of new drugs [7]. Propolis has been widely used in folk medicine and has shown promising results against some protozoonoses [8].

The chemical composition of propolis is dependent on the biodiversity of each area visited by the bees, and the quantity of biologically active compounds present in each sample may change [7]. Herein, our propolis sample was collected in apiary of UNESP, Campus of Botucatu, Brazil, which has not been previously assessed for its antileishmanial action. Our group verified that this sample induced an anti-inflammatory response affecting CCL5 and IFN-*γ* expression in peripheral blood mononuclear cells in both healthy individuals and leishmaniasis patients [9]. Besides, previous studies have shown the antiprotozoal activity of different propolis extracts, demonstrating the leishmanicidal action on both promastigotes and amastigotes forms of different *Leishmania* spp. [10, 11].

Based on these observations, the goal of this study was to evaluate the antileishmanial and immunomodulatory properties of propolis on the experimental infection with *L. braziliensis*. 

## 2. Material and Methods

### 2.1. *Leishmania (Viannia) braziliensis*

*Leishmania (Viannia) braziliensis* (MHOM/BR/1987/M11272) was used in promastigote forms, kept in culture medium 199 (GIBCO Invitrogen), and supplemented with 10% fetal bovine serum (FBS-GIBCO Invitrogen), 1 M Hepes, 0.1% human urine, 0.1% L-glutamine, 10 *μ*g/mL penicillin and streptomycin (GIBCO Invitrogen), and 10% sodium bicarbonate (complete medium for promastigotes—CMP). Cell cultures were incubated at 25°C in 25 cm^2^ flasks.

### 2.2. Sample Propolis

Propolis sample was collected in the Beekeeping Section of the Lageado Farm, UNESP, Campus of Botucatu, Brazil, from honeybee (*Apis mellifera* L.) colonies. The method of extraction and its chemical composition can be seen in previous works of our group [12].

### 2.3. Kinetics of Cellular Proliferation

Promastigote forms (1 × 10^6^/mL) incubated in CMP were treated with different concentrations of propolis (5, 10, 25, 50, and 100 *μ*g/mL) and Glucantime (250 *μ*g/mL CMP) or with propolis solvent (0.1% ethanol/mL) and cultured for 7 days at 25°C. Promastigotes were counted in a Neubauer chamber after 24, 96, and 168 hours (h). As control, the culture medium alone and propolis solvent were used.

### 2.4. Scanning Electron Microscopy

Promastigotes (2 × 10^6^/mL) in exponentially growing phase were cultured in 199 medium with propolis (5, 10, 25, 50, and 100 *μ*g/mL) for 2 or 24 h at 25°C in sterile Falcon tubes. As control, only the culture medium (CMP) alone and 0.1% ethanol were used. Afterwards, promastigotes were centrifuged twice at 600 g for 10 minutes and resuspended in 0.9% sterile saline solution pH = 7.0. Promastigotes were placed on glass coverslips covered with Poly-L-lysine (GIBCO), fixed with 2% glutaraldehyde in phosphate buffer 0.1 M (pH = 7.0), and postfixed with 1% osmium tetroxide. Dehydration was performed with ethanol (70, 80, 90, and 100°GL). The critical point was carried out with a *Critical Point Dryer* (PCD 030—BAL-TEC) on stubs for recovery with 30 nm gold, using a *Stutter coater *(SCD 050—BAL-TEC). Samples were analyzed using an FEI QUANTA 200 scanning electron microscope (SEM).

### 2.5. Animals

Male BALB/c mice weighing approximately 25–30 g and aged 6–8 weeks old were obtained from the Medical School of Ribeirão Preto, USP, Brazil. Mice were kept under pathogen-free conditions and used according to protocols approved by the institutional Animal Care. This work was approved by the Londrina State University—Ethics Committee for Animal Experimentation (n. 09/2011).

### 2.6. Phagocytic Assay

Macrophages (5 × 10^5^/mL) were obtained from the peritoneal cavity by the injection of 2 mL of RPMI 1640 culture medium (GIBCO) supplemented with fetal bovine serum 10% (GIBCO) and cultured on 24-well plates containing 13 mm diameter glass coverslips. Cells were preincubated with 200 *μ*L RPMI medium for 2 h for adherence, treated with propolis (5 or 10 *μ*g/mL) or with RPMI medium alone (control), and incubated for 24 h at 37°C and 5% CO_2_. After, macrophages were washed and infected with promastigotes forms (5 : 1) for 2 h. Cells were stained with Giemsa to establish the phagocytic index by means of % of infection and the parasites/macrophages number. 

In another protocol, peritoneal macrophages (5 × 10^5^/mL) were cultured on 6-well plates at 37°C and 5% CO_2_ with 5 or 10 *μ*g/mL of propolis. After 24 h, macrophages were washed and infected with promastigotes forms (5 : 1) for 2 h. The culture was washed to remove extracellular parasites and incubated with 199 culture medium at 24°C. Recovered promastigotes were counted in a Neubauer chamber 3 days after the infection.

### 2.7. Cytokine Determination

Mice were treated intraperitoneally with propolis (2.5, 5, or 10 mg/kg) or solvent only (ethanol 0.1%) [13, 14]. After 24 h, mice were infected intraperitoneally with 10^7^/mL promastigote forms of *L. braziliensis*. Mice were sacrificed 2 h after infection, and the exudate cells were collected by rinsing the peritoneal cavity with 2 mL of RPMI medium. Cells were distributed on 6-well plates for 1 h at 37°C and 5% CO_2_. Supernatants were collected, centrifuged at 460 g at 4°C for 7 min, and stored at −20°C for cytokine determination. The liver was removed, macerated, centrifuged, and stored at −20°C for IL-12 and TNF-*α* levels determination by enzyme-linked immunosorbent assay (ELISA), according to manufacturer's instructions (eBiosciencesR, USA). Plates were read at 450 nm, using an ELISA plate reader (Thermo Plate—TP-Reader).

### 2.8. Statistical Analysis

Data were analyzed using the Prism GraphPad statistical software (GraphPad Software, Inc., USA, 500.288). Significant differences between treatments were determined by ANOVA, followed by the Tukey test for multiple comparisons. Statistical significance was accepted when *P* < 0.05.

## 3. Results

### 3.1. Propolis Effect on *L. braziliensis* Promastigotes Proliferation

**Figure 1:**
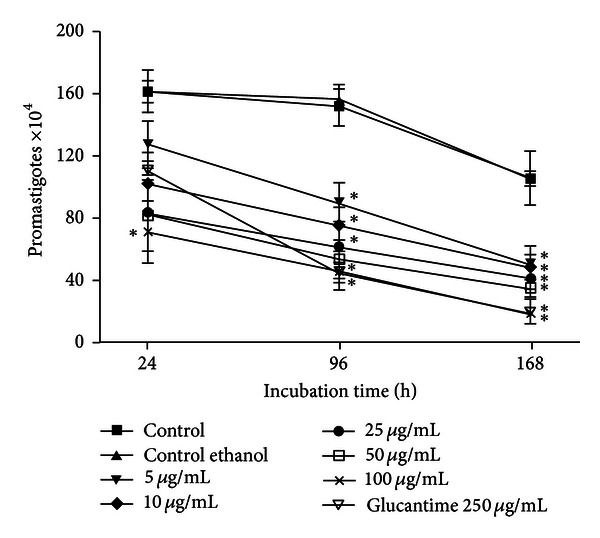
Kinetics of *L. braziliensis *promastigotes proliferation after treatment with propolis (5, 10, 25, 50, and 100 *μ*g/mL) or Glucantime for 24, 96, and 168 h. Data represent mean ± SEM of five independent experiments. *Significantly different from control (*P* < 0.05).

Figure 1 shows that propolis prevented cell proliferation using 100 *μ*g/mL for 24 h (*P* < 0.05). After 96 and 168 h, propolis in all concentrations reduced cell proliferation (*P* < 0.05). Propolis (100 *μ*g/mL) showed the same antiproliferative effect of Glucantime (250 *μ*g/mL) after 96 and 168 h of incubation. The solvent (0.1% ethanol/CMP) did not interfere in promastigotes *L. braziliensis* proliferation. 

### 3.2. Scanning Electron Microscopy (SEM)

**Figure 2:**
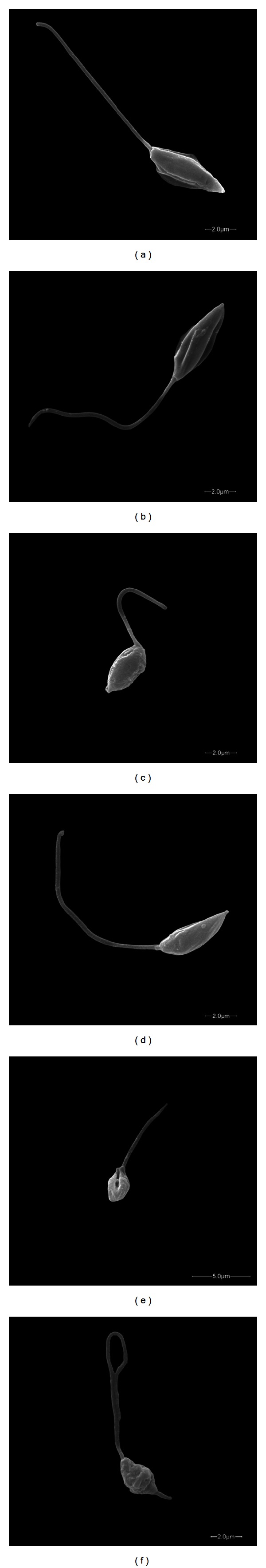
Scanning electron microscopy showing promastigotes forms of *L. braziliensis *treated with different concentrations of propolis by 24 h. (a) Control; (b) 5 *μ*g/mL; (c) 10 *μ*g/mL; (d) 25 *μ*g/mL; (e) 50 *μ*g/mL; (f) 100 *μ*g/mL.

*L. braziliensis *treated with propolis (10, 25, 50 and 100 *μ*g/mL) showed morphological changes. After 2 h these changes were similar to those observed after 24 h of incubation, and some cells presented signs of shrinkage whereas others exhibited flagella with several lengths. The images shown in Figure 2 represent the overview changes found in each concentration. Propolis solvent (0.1% ethanol) did not interfere on the morphology of promastigotes forms of *L. braziliensis* (data not shown). 

### 3.3. Phagocytic Assay

**Figure 3:**
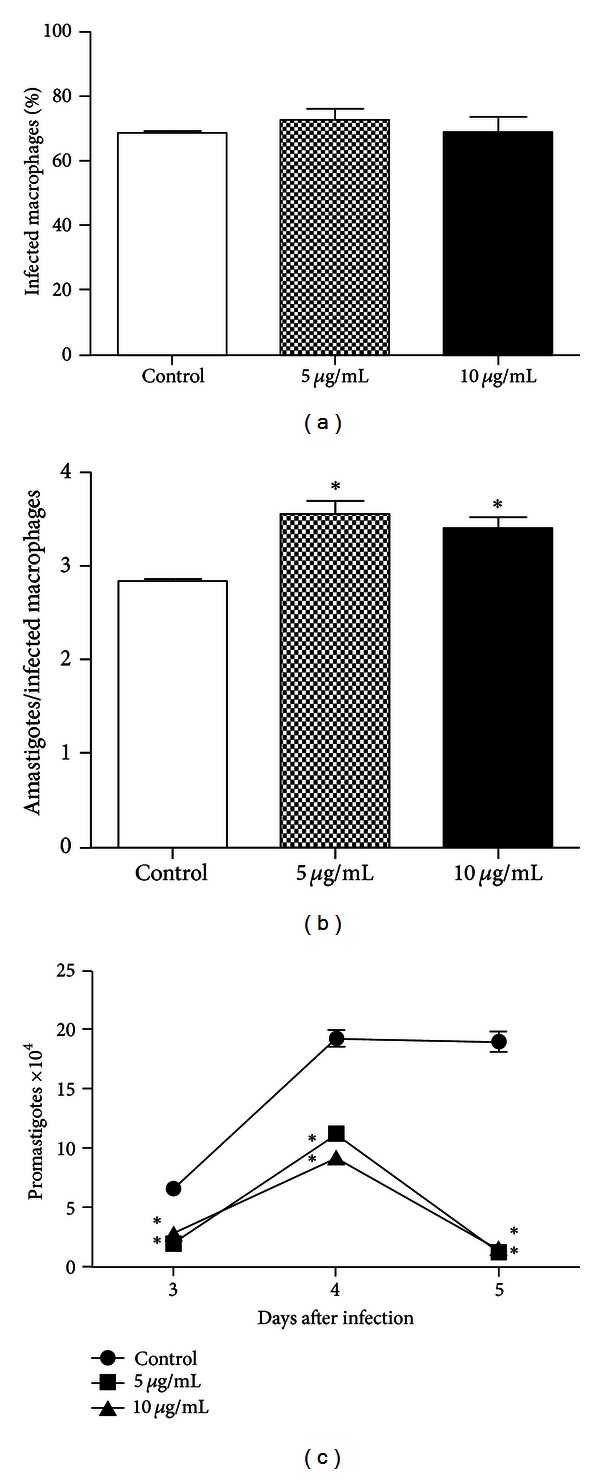
Propolis effect *in vitro *on *L. braziliensis* phagocytosis by macrophages. Monolayers of macrophages were treated with propolis (5 or 10 *μ*g/mL) or with RPMI culture medium (control), incubated for 24 h at 37°C, and then coincubated for 2 h with promastigotes forms. (a) Percentage of macrophages phagocytosis; (b) number of intracellular amastigotes per macrophage after 2 h; (c) kinetics of recovered promastigotes from infected macrophages. Data represent mean ± SEM of three independent experiments. *Significantly different from control (*P* < 0.05).

As shown in Figure 3(a), no significant differences were seen in the percentage of infected macrophages incubated with propolis in comparison to control. However, the mean of amastigotes number per macrophage was significantly increased after 2 h using 5 and 10 *μ*g/mL (Figure 3(b)). In an attempt to verify the capacity of propolis to induce the activation of macrophages to eliminate intracellular parasites, a reduction in recovered promastigotes was seen in propolis-treated cells 3 days after infection, reaching 82% and 94.4% with 5 and 10 *μ*g/mL propolis, respectively, at the fifth day (Figure 3(c)).

### 3.4. Cytokine Determination

**Figure 4:**
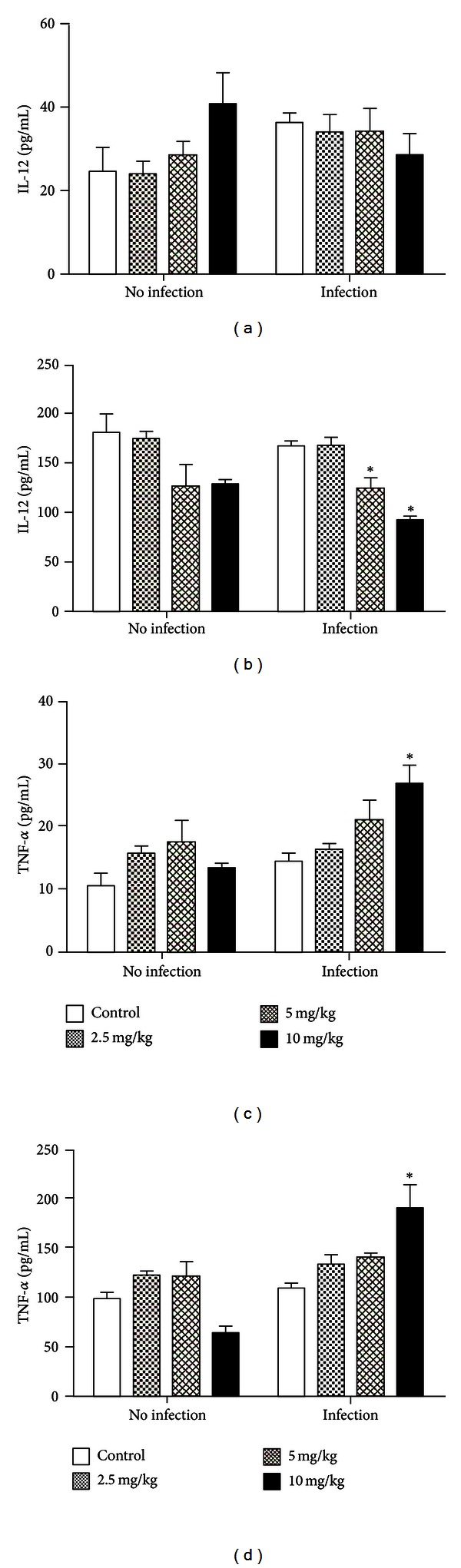
IL-12 and TNF-*α* production (pg/mL) by peritoneal macrophages and liver cells from propolis-treated mice (2.5, 5, and 10 mg/kg), infected or not with *L. braziliensis* for 2 h. (a) IL-12 production by peritoneal exudate; (b) IL-12 production by liver cells; (c) TNF-*α* production by peritoneal exudate; (d) TNF-*α* production by liver cells. Data represent mean ± SEM of three independent experiments. *Significantly different from control (*P* < 0.05).

Propolis treatment (2.5 mg/kg: 23.86 pg/mL ± 3.09; 5 mg/kg: 28.66 pg/mL ± 3.02; 10 mg/kg: 40.73 pg/mL ± 7.63) did not affect IL-12 production by peritoneal macrophages in comparison to control (25.01 pg/mL± 5.59) (*P* > 0.05), even associated to infection (control: 36.77 pg/mL ± 2.29; propolis 2.5 mg/kg: 34.05 pg/mL ± 4.25; 5 mg/kg: 34.37 pg/mL ± 5.25; 10 mg/kg: 28.73 pg/mL ± 5.01) (Figure 4(a)).

Likewise, propolis treatment did not alter IL-12 production in the liver (2.5 mg/kg: 174.42 pg/mL ± 6.18; 5 mg/kg: 126.58 pg/mL ± 21.74; 10 mg/kg: 128.34 pg/mL ± 4.04), in comparison to control (182.35 pg/mL ± 17.65) (*P* > 0.05). However, associated to infection, IL-12 levels were lower in mice pretreated with 5 mg/kg (124.62 pg/mL ± 9.54) and 10 mg/kg (92.55 pg/mL ± 3.18) compared to control (168.79 pg/mL ± 3.17) (*P* < 0.05) (Figure 4(b)). 

No alterations were seen without infection in TNF-*α* secretion by macrophages (control: 10.75 pg/mL ± 1.98; propolis 2.5 mg/kg: 15.44 pg/mL ± 1.26; 5 mg/kg: 17.65 pg/mL± 3.22; 10 mg/kg: 13.15 pg/mL ± 0.92) or in the liver (control: 99.39 pg/mL ± 6.64; propolis 2.5 mg/kg: 122.19 pg/mL ± 3.10; 5 mg/kg: 122.03 pg/mL ± 13.61; 10 mg/kg: 64.12 pg/mL ± 7.17) (*P* > 0.05). However, TNF-*α* production was significantly increased (*P* < 0.05) in peritoneal exudate (26.84 pg/mL± 2.91) and liver homogenate (190.30 pg/mL ± 23.90) from propolis-treated mice (10 mg/kg) after infection (Figures 4(c) and 4(d)).

## 4. Discussion

The plants most frequently visited by bees in the apiary located at UNESP, Campus of Botucatu, were *Baccharis dracunculifolia*, *Eucalyptus citriodora*, and *Araucaria angustifolia *[12]. Besides the species of plants visited by bees to produce propolis, the chemical composition of propolis may be influenced by the solvent as well by genetic characteristics shown by different bee subspecies [15–17]. The major components of our propolis sample were phenolic compounds (flavonoids, aromatic acids, and benzopyranes), di- and triterpenes, and essential oils. Phenolic compounds have been related to antimicrobial, trypanocidal, and antitumoral activities [18]. 

In experimental leishmaniasis, the compounds associated with the inhibition of amastigotes proliferation were prenylated and benzophenones [10]; caffeic acid, *p*-coumaric acid, aromadendrine-4′-methyl ether, 3-prenyl-*p*-coumaric, and 3,5-diprenyl-*p*-coumaric exerted a direct effect on promastigote forms and decreased lesion after infection *in vivo *[19]; flavonoids, monosaccharaides, and other phenolic compounds were involved in the antileishmanial action in promastigote forms of *L. major, L. chagasi*, and *L. braziliensis *[20]. These compounds have been found in high quantities in different propolis samples and have been associated with results obtained in experimental leishmaniasis, suggesting that the propolis chemical composition is one of the main factors to be investigated to evaluate its pharmacological activities. However, a synergistic effect presented by different compounds should not be discarded when assessing its biological effects.

 In this study, a direct effect of propolis preventing promastigote proliferation was verified, which was significant at a small dose (5 *μ*g/mL) after 96 h of incubation. Moreover, the highest dose (100 *μ*g/mL) showed a similar efficacy to Glucantime 250 *μ*g/mL in all periods of times. Morphologic alterations such as shrinkage of promastigotes were observed by scanning electron microscopy from 10 *μ*g/mL. Taken together, these results suggested that our propolis sample exhibited a leishmanicidal activity.

 To determine the effects of treating macrophages with propolis before infection with *L. braziliensis, *the concentrations of 5 and 10 *μ*g/mL were used, since higher doses showed cytotoxic effects in human cells [9]. There was a significant increase in parasite interiorization by macrophages pretreated with 5 or 10 *μ*g/mL compared to control, which is in agreement with Orsatti et al., who found increased Toll-like receptors (TLR)-2 and TLR-4 expression in macrophages from BALB/c mice pretreated with propolis [21]. Besides these receptors, it has been described that other receptors are involved in *Leishmania* recognition, such as complement receptor (CR) 1, CR3, and mannose receptor [22]. Furthermore, after 3 days of coculture, the amount of recovered promastigotes was reduced significantly in cells treated with 5 and 10 *μ*g/mL, showing that propolis upregulated macrophage microbicidal mechanisms.

 Cytokines have an important role in the clinical outcome of leishmaniasis, activating macrophages and its phagocytic activity in the early stages of infection [23, 24]. In our assays, IL-12 and TNF-*α* production was evaluated after BALB/c mice treatment with propolis and infection with *L. (V.) braziliensis. *IL-12 concentration was reduced in the liver during infection, whereas TNF-*α* production increased significantly in both peritoneal exudate and liver cells compared to noninfected mice. Studies have shown that some species of *Leishmania *can inhibit IL-12 synthesis through the PI3K pathway, which may not interfere with the production of other cytokines such as TNF-*α* [25, 26]. Moreover, it was verified that *L. donovani *modulate TLR-2 by suppressing MAPK P38 phosphorylation with a consequent reduction of IL-12 production [27]. After propolis administration to mice, an increased TLR-2 was seen in peritoneal macrophages [21]. However, although this is an important pathway for signaling IL-12 production, in this study *L. brazilienzis *reduced this cytokine production. On the other hand, TLR-4, which is involved in TNF-*α* production, was significantly increased in propolis-treated mice compared to control [21], with no parasite interference, leading to a higher ingestion, TNF-*α* production, and leishmanial activity by macrophages.

 In conclusion, propolis showed a direct action on the parasite and displayed immunomodulatory effects on murine macrophages, even though the parasite has been reported to affect these activation pathways of the cell.
